# Supplementary material for: Education and Information to Improve Adherence to Screening for Breast, Colorectal, and Cervical Cancer—Lessons Learned during the COVID-19 Pandemic
Source: Cancers (Basel). 2024 Aug 31;16(17):3042. doi: 10.3390/cancers16173042 (PMC11394549; doi:10.3390/cancers16173042)
Supplement: Supplementary file 1 [file cancers-16-03042-s001.zip › CANCERS SUPPLEMENT TABLE s1.pdf]

SUPPLEMENT TABLE S 1 Pathology of Breast Cancer : 9706 patients who did not receive pre-operative neoadjuvant. Analysis by the GIPAD* and by the SIAPC**(12 Large Volume Hospitals)							
	2019	%Total 2019	2020	%Total 2020	2021	%Total 2021	
N. Patients	3.189		3.083		3.479		
pT s+ pT1	1169	73.4%	1040	72.8%	1172	72.9%%	
pT2	717	22.5%	698	23.0%	785	22.6%	
pT3	79	2.5%	85	2.8%	113	3.2%	
pT4	31	1.0%	43	1.4%	46	1.3%	
pN0	2.442	76.6%	2.319	76.3%	2.671	76.8%	
pN1	554	17.4%	514	16.9%	618	17.8%	

pN2	117	3.7%	104	3.4%	95	2.7%	
pN3	76	2.4%	101	3.2%	95	2.7%	
*Gruppo Italiano Patologia Apparato Digerente **Società Italiana Anatomia Patologica e Citologia.							
