# Supplementary material for: Education and Information to Improve Adherence to Screening for Breast, Colorectal, and Cervical Cancer—Lessons Learned during the COVID-19 Pandemic
Source: Cancers (Basel). 2024 Aug 31;16(17):3042. doi: 10.3390/cancers16173042 (PMC11394549; doi:10.3390/cancers16173042)
Supplement: Supplementary file 1 [file cancers-16-03042-s001.zip › table S 3 cancers Country cRC mortality (1).pdf]

SUPPLEMENT TABLE s3.

MORTALITY RATES FROM COLORECTAL CANCER FOR INDIVIDUALS 50-74 YEARS OF AGE *

Country	2010 all 50-74	2010 F Women	2010 Men	2020 all 50-74	2020 Women	2020 Men
BELGIUM	44.8	31.4	59.1	31.8	25.5	38.5
AUSTRIA	46.7	34.1	60.6	33.1	22.9	43.9
GERMANY	52.4	38.0	67.9	39.2	28.1	51.0
FRANCE	43.3	31.4	56.4	37.1	27.8	47.2
DENMARK	64.4	52.0	77.1	42.3	35.2	49,.6
IRELAND	48.9	36.2	61.9	41.6	31.4	52.1
ICELAND	33.4	18.6	48.2	46.1	43.6	48.7
UK	46.8	33.5	61.0	41.1	32.7	50.0
ITALY	45.7	32.1	58.7	34.2	24.4	44.3
SPAIN	56.9	36.1	79.9	45.1	30.3	61.2
PORTUGAL	62.5	39.3	89.9	50.0	34.3	68.5
GREECE	31.4	22.8	41.0	35.5	25.2	47.3
MALTA	58.1	48.4	68.5	42.2	31.2	53.4
CROATIA	84.6	52.0	122.8	82.8	51.8	118.1
CZECHIA	79.1	50.4	112.1	60.4	40.0	83.0
LATVIA	70.7	58.3	89.3	56.9	42.4	76.8
LITHUANIA	65.0	46.3	92.0	50.6	36.0	70.4
FINLAND	34.7	26.8	43.1	36.3	29.5	43.6
ESTONIA	62.5	46.4	85.5	45.9	31.3	64.8
BULGARIA	68.3	47.5	93.7	71.6	47.6	100.3
HUNGARY	107.5	74.4	151.0	103.7	69.4	147.0
SWITZERLAND	32.3	23.2	40.8	22.2	17.4	26.8

*Data from Institute for Health Metrics and Evaluation (IHME). GBD Compare Data Visualization. Seattle, WA: IHME, University of Washington, 2024. Available from http://vizhub.healthdata.org/gbd-compare(link is external). (Accessed, 10th August 2024).)
