# Supplementary material for: Education and Information to Improve Adherence to Screening for Breast, Colorectal, and Cervical Cancer—Lessons Learned during the COVID-19 Pandemic
Source: Cancers (Basel). 2024 Aug 31;16(17):3042. doi: 10.3390/cancers16173042 (PMC11394549; doi:10.3390/cancers16173042)
Supplement: Supplementary file 1 [file supplement table s 2 (3).pdf › supplement table s 2 (3).pdf]

**SUPPLEMENT TABLE s2 Pathology of Colorectal Cancer: 8287 patients who did not receive pre-operative neoadjuvant. Analysis by the GIPAD* and by the SIAPC****							
	2019	%Total 2019	2020	%Total 2020	2021	%Total 2021	
N. Patients<br><br>N. Involved Centers	3.146<br><br>19		2.952<br><br>19		2.189<br><br>12		
T STAGE							
pT in situ	128	3.7%	86	2.9%	199	9.1%	
pT1	300	8.8%	282	9.6%	240	11.0%	
pT2	488	14.3%	426	14.4%	338	15.4%	
pT3	1.814	53.1%	1.498	50.7%	977	44.6%	
pT4	686	20.1%	660	22.4%	435	19.9%	
LYNPHONODE STAGE							
pN0	2.062	60.4%	1.756	59.5%	1.444	66.0%	
pN1	868	25.4%	737	24.9%	484	22.1%	
pN2	256	7.5%	235	8.0%	134	6.1%	
pN3	230	6.7%	224	7.6%	127	5.8%	
*Gruppo Italiano Patologia Apparato Digerente **Società Italiana Anatomia Patologica e Citologia.							
